# Epigenetic variability in conversion to psychosis: novel findings from an innovative longitudinal methylomic analysis

**DOI:** 10.1038/s41398-018-0138-2

**Published:** 2018-04-26

**Authors:** Oussama Kebir, Boris Chaumette, Marie-Odile Krebs

**Affiliations:** 1 Centre de Psychiatrie et Neurosciences, Université Paris Descartes, PRES Université Paris Sorbonne Paris Cité, UMR S 894 Paris, France; 2 Laboratoire de Physiopathologie des Maladies Psychiatriques, Centre de Psychiatrie et Neurosciences, INSERM, UMR S 894 Paris, France; 3 CNRS, GDR3557-Institut de Psychiatrie, Paris, France; 4 Faculté de Médecine Paris Descartes, Centre Hospitalier Sainte-Annes, Service d’Addictologie «Moreau de Tours», Paris, France; 5 Faculté de Médecine Paris Descartes, Centre Hospitalier Sainte-Anne, Service Hospitalo-Universitaire, Paris, France

## Abstract

Conversion to psychosis is a longitudinal process during which several epigenetic changes have been described. We tested the hypothesis that epigenetic variability in the methylomes of ultra-high risk (UHR) individuals may contribute to the risk of conversion. We studied a longitudinal cohort of UHR individuals (*n* = 39) and compared two groups (converters, *n* = 14 vs. non-converters, *n* = 25). A longitudinal methylomic study was conducted using Infinium HumanMethylation450 BeadChip covering half a million cytosine–phosphate–guanine (CpG) sites across the human genome from whole-blood samples. We used two statistical methods to investigate the variability of methylation probes. (i) The search for longitudinal variable methylation probes (VMPs) based on median comparisons identified two VMPs in converters only. The first CpG was located in the *MACROD2* gene and the second CpG was in an intergenic region at 8q24.21. (ii) The detection of outliers using variance analysis related to private epimutations identified a dozen CpGs in converters only and highlighted two genes (*RAC1* and *SPHK1*) from the sphingolipid signaling pathway. Our study is the first to support increased methylome variability during conversion to psychosis. We speculate that stochastic factors could increase DNA methylation variability and have a role in the complex pathophysiology of conversion to psychosis as well as in other psychiatric diseases.

## Introduction

Over the past two decades, the concept of psychosis has moved from a chronic presentation to a more dynamic paradigm. Accordingly, schizophrenia is now conceptualized as a progressive illness that typically emerges during late adolescence with transitions across several stages: early vulnerability, at-risk mental state (also called ultra-high risk, abbreviated UHR), first episode of psychosis, and chronic disease. This new conception raises hope that earlier treatment could prevent the emergence of psychosis. The UHR state refers to individuals with prodromal symptoms who may (or may not) develop full-blown psychosis. Overall, 30–40% of UHR individuals convert to full-blown psychosis in the following 24 to 36 months^1^.

Complex diseases, such as cancer or psychiatric disorders, result from interactions between genes and the environment. Epigenetic regulations mediate this interplay, at least partly. The epigenome refers to the biological mechanisms, which regulate gene expression, including DNA methylation. The epigenome is stable overall, but it can be altered by environmental factors, or by stochastic mechanisms^2^. Though understanding the mechanisms leading to epigenome stability or instability has just begun, it may be of particular medical interest. Increased DNA methylation variability has been shown to affect carcinogenesis^3,4^ and may also be involved in obesity^5^, major depression disorder^6,7^, or depressive symptoms^8,9^.

Two mechanisms of methylomic variability are open to molecular investigation. First, methylomic variability could be located in specific genomic regions, as specific variably methylated probes (VMPs) have been identified through median comparisons. VMPs have previously been identified in association with depressive symptoms^9^. The second mechanism refers to private epimutations, i.e., over time, ‘individual-specific methylation changes’ may occur in some individuals only. These epimutations are most likely due to stochasticity. In fact, during cell replication, the methylome is less preserved than the genome and modifications randomly occur at a rate of 1/10^3^ (vs. 1/10^6^ with genomic replication)^10^. Notably, a stochastic epigenetic model of cancer has been described as a mechanistic explanation for tumor cell heterogeneity^3^.

Previously, we have demonstrated that longitudinal methylomic changes could be involved in conversion to psychosis. Inter-group differences revealed specific dysmethylation in genes (involved in redox metabolism, axonal guidance, and inflammation in UHR individuals) who converted to psychosis (‘converters’)^11^. We also found evidence for heterogeneity and hypothesized that intra-individual differences during follow-up could be associated with disease progression in some participants. The present work aims to describe individual DNA methylation variability in the same dataset, during conversion to psychosis. The longitudinal design offers the benefit of suppressing potential sources of methylation variability due to DNA sequence variability by comparing individuals to themselves. Moreover, the 1-year follow-up enabled to explore short-term, longitudinal methylomic variability associated with the emergence of psychosis. To investigate the variability of methylation probes during conversion to psychosis, we propose to use two statistical methods: (i) searching for longitudinal VMPs based on median comparisons, and (ii) detecting outliers with beta-value variance analysis related to private epimutations.

## Materials and methods

### Population

The study was approved by the Institutional ethics committee ‘Comité de protection des personnes, Ile-de-France III, Paris, France’. Written informed consent was obtained from all participants in accordance with the Declaration of Helsinki. Help-seeking individuals (16–30 years) consecutively referred to the Adolescent and Young Adult Assessment Centre (Service Hospitalo-Universitaire, Hôpital Sainte-Anne, Paris, France) between 2009 and 2013 were enrolled in the ICAAR collaborative study promoted by Sainte-Anne Hospital as described^12^ previously. Follow-up lasted a year. Inclusion criteria were global functioning alterations (Social and Occupational Functioning Assessment Scale score <70) during the past year, associated with psychiatric symptoms and/or subjective cognitive complaints. All participants were examined by specifically trained psychiatrists, using a translated French version^13^ of the Comprehensive Assessment for at-risk mental states (CAARMS^14^). Best-estimate diagnoses were allocated at a subsequent consensus meeting. The CAARMS UHR definition is based on the presence of sub-threshold positive psychotic symptoms (e.g., ideas of reference, ‘magical’ thinking, perceptual disturbance, paranoid ideation, odd thinking and speech) with either sub-threshold frequency (<3 times per week) or sub-threshold intensity (attenuated psychosis syndrome). Participants who reached the CAARMS-based psychosis clinical threshold during follow-up were classified as converters (*n* = 14, i.e., marked thought content disorders, perceptual abnormalities and/or disorganized speech with a frequency of more than three to six times per week, over an hour each time and present for longer than one week). UHR subjects who did not reach psychosis threshold were classified as non-converters (*n* = 25). Exclusion criteria included conspicuous symptoms of psychosis, pervasive developmental or bipolar disorders, and individuals with other established diagnoses, such as obsessive-compulsive disorder (based on the Diagnostic and Statistical Manual of Mental Disorders, Fourth Edition). Other exclusion criteria were: current antipsychotic treatment (>100 mg Chlorpromazine equivalent) for >12 weeks, psychoactive substance dependence or abuse in the previous year and/or >5 years, severe or non-stabilized somatic and neurological disorders, head injury and intelligence quotient <70. Whole blood was sampled at inclusion (M0) and after one year, or after psychosis onset (MF). Potential confounding factors, such as sex ratio, age, follow-up duration, body mass index, substance abuse, and psychotropic treatment introduction were recorded. None of these variables was significantly different between groups (converters vs. non-converters; Table 1). Substance abuse was not significantly different between M0 and MF. No change in alcohol or tobacco use was reported in any individual. Two converters quit using cannabis and one converter started using cannabis.

**Table 1:** Sample characteristics

	Converters	Non-converters	Significance
	*n* = 14	*n* = 25	
Clinical variables mean (s.d.)			
Sex ratio (M/F)	9/5	13/12	*p* = 0.52_a_
Age	21.9 (3.6)	23.8 (4.1)	*p* = 0.17^b^
Body mass index	20.9 (3.5)	21.9 (4.7)	*p* = 0.66^b^
Follow-up			
Biological interval in months	10.1 (7.2)	11.4 (5.8)	*p* = 0.62^b^
Clinical follow-up in months	10.7 (7)	12.7 (5.7)	*p* = 0.22^b^
Substance use (user/non-user)			
Lifetime cannabis use	7/7	5/20	*p* = 0.07^a^
Alcohol use (once a week during 6 months)	6/8	13/12	*p* = 0.74^a^
Daily/regular tobacco use	7/7	10/15	*p* = 0.39^a^
Psychotropic treatment during follow-up			
Antipsychotic or valproate introduction	6/8	4/21	*p* = 0.12^a^
Other psychotropic medication introduction	4/10	2/23	*p* = 0.16^a^

No significant differences were identified for potential confounding factors. Biological interval represents time between the two blood samples. Clinical follow-up represents time between inclusion and final status assessments

*F* female, *M* male

^a^ *p* is given by Fisher’s test

^b^ *p* is given by non-parametric Mann–Whitney test

### Genome-wide analysis of DNA methylation

#### Preparation

For each individual (*n* = 39; two times of assessment), genomic DNA (500 ng) was extracted from whole blood using the Wizard ® genomic DNA purification kit (TM050, Promega, USA), which preserves DNA methylation. Then, the DNA was treated with sodium bisulfite using the EZ-96DNA Methylation KIT (Catalog No D5004, Zymo Research, Irvine, CA, USA) following the manufacturer’s standard protocol. Methylation was measured in all samples simultaneously, using the same technique. Genome-wide DNA methylation was assessed using Illumina Infinium HumanMethylation450 BeadChip (Illumina, San Diego, CA, USA), which interrogates the DNA methylation profile of >485,000 CpG loci across the genome at single-nucleotide resolution. This chip explores all the known genes (according to UCSC). This technique is reliable compared to others, like pyrosequencing^15^, and we previously showed strong correlation between our data from the chip and those obtained using pyrosequencing^11^.

#### Data preprocessing and clean up

GenomeStudio software (Illumina) was used to extract signal intensities in each probe. All computations and statistical analyses were conducted in the R statistical analysis environment. All the scripts are available (from the authors) on request. R methylumi package was used for data quality check, including gender check between phenotype file and methylation dataset, evaluation of concordance between the two samples from each individual, flagging and removing individuals with no result, gender discrepancies, or discordant genotypes, and probes with beadcount <3 in ≥5 % of samples. Samples with >4500 CpG (1%) with a detection *p*-value ≥ 0.05 are more subject to methodological bias and should be excluded from analysis. No sample was excluded. Probes with a detection *p*-value ≥ 0.05 in one sample at least were excluded from analysis. Additionally, probes on chromosomes X and Y, SNP probes, probes with a SNP at the CpG site, and cross-reactive probes that map to more than one location in the genome were removed. The final methylation data file includes 411,947 probes. No sample was removed in the process of quality check. R package minfi was used for normalization using the FunNorm function that provides the beta-value (*β*). Then the beta-values were adjusted on the mixture blood cell counts using the EstimateCellCounts function. All the samples were processed and analyzed at the same time. Batch effect, e.g., plate or slide, was systematically screened using a surrogate variable analysis implemented in the Combat R Package (SVA function). Neither batch effect nor unaddressed confounding factor was detected. R scripts are provided in the supplementary material.

### Methylomic variability analysis

Two methods were used with beta-values.

#### Detection of variably methylated probes (VMPs)

**Fig. 1:**
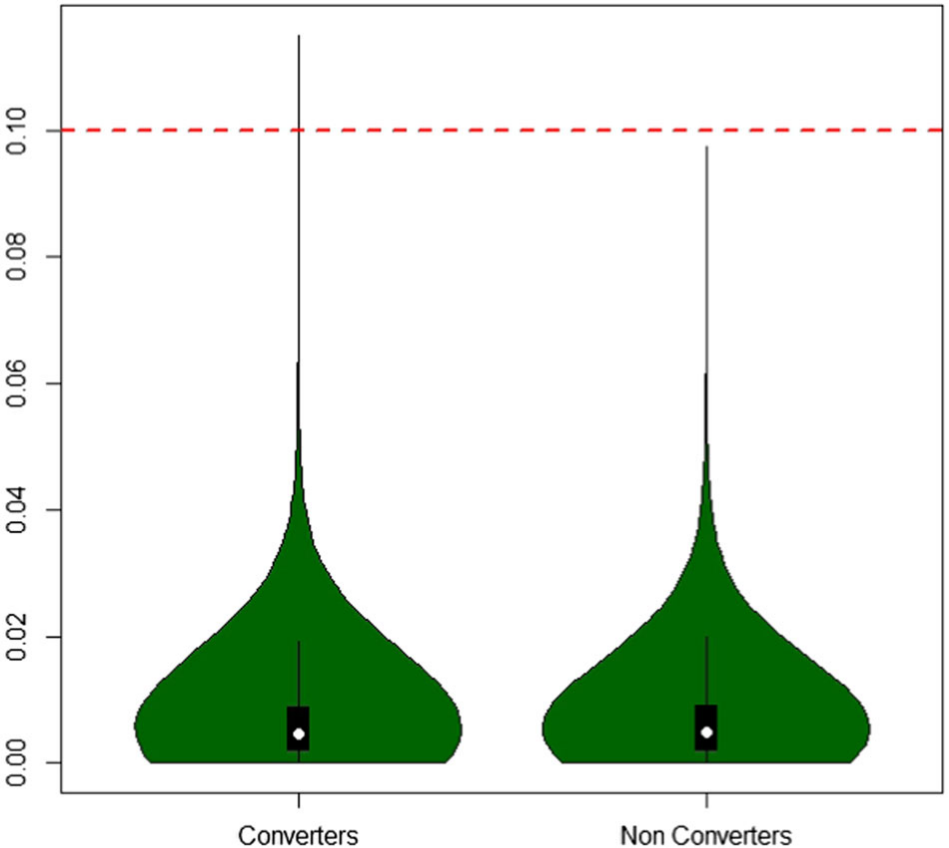
Violin plot showing the repartition of |median(βΔ)| in converters and non-converters. The probes with a value >0.10 are considered to be variably methylated probes

This method is based on an analytic approach of centrality measures and was used to compare methylomes of concordant and discordant twins for depressive symptoms to healthy twins^9^. Given the context, the method was adapted for a longitudinal design in which individuals were compared to themselves at various moments. Thus, longitudinal differences in blood sample DNA methylation levels Δ*β* = *β*(MF) – *β*(M0) were computed for all CpG sites across the genome. Then, the median value of absolute DNA methylation differences |median(Δ*β*)| was computed for both outcome groups (converters and non-converters). On the basis of the distribution of the |median(Δ*β*)| (Fig. 1) and on previous reports indicating that methylation differences above 10% in Illumina assays have biological significance and low probability of being technical artifacts^16,17^, we filtered out CpG sites with <10% difference from the median group (Δ*β*). In other words, a CpG site was considered ‘variable’ if the median (Δ*β*) was greater than or equal to the absolute value of 0.1.

#### Detection of private epimutation

This method is based on variance difference in each probe comparing two groups as examined in^5,6,8,18^. Independent *F*-tests were conducted in each CpG in our experimental context to compare converters to non-converters at M0 and at MF. *F*-test is reputed to be very sensitive to significant differences in variances between two groups. For each CpG, the null hypothesis was equality of beta-value variances between converters and non-converters. Multiple testing adjustments were conducted using the false discovery rate (FDR). Significance was reached for a two-sided adjusted-*p* value below a 0.05 threshold. Probes with significant differences in variance between converters and non-converters at MF were selected as potential private epimutations. These epimutations were considered clinically relevant in conversion to psychosis if: (i) the probes displayed greater variance in MF compared to M0 (variance increases with time), (ii) the probes were identified only in converters, (CpG more heterogeneous in converters than non-converters), and (iii) individual variance deviated more than 10% from the median group (again, the 10% cut-off was based on previous references^7,17^).

### Annotation and over-representation analyses

All the chromosomal annotations were based on hg19 genome version. Genes are named by Illumina Annotation File (UCSC gene name). Over-representation analyses were conducted in clinically relevant CpGs using EnrichR^19^ which draws upon standard databases (KEGG, Reactome, and Gene Ontology) containing all known genes with UCSC annotation. The brain-expressed genes were also submitted to bibliographic search in Pubmed and OMIM.

## Results

### Identification of longitudinal regional variability: variably methylated probes (VMPs)

We focused on CpGs with large longitudinal methylation differences (absolute difference >10%). These CpGs were supposed to tag regions with methylomic instability. Using these criteria, two probes were identified in converters: one CpG was located in the MACROD2 gene and one was located at 8q24.21 (chr8:129702875). The median Δβ was 0.11 for both CpGs. By contrast, no VMPs were detected in non-converters (Fig. 1).

### Identification of longitudinal individual variability (private epimutations): outlier detection

**Fig. 2:**
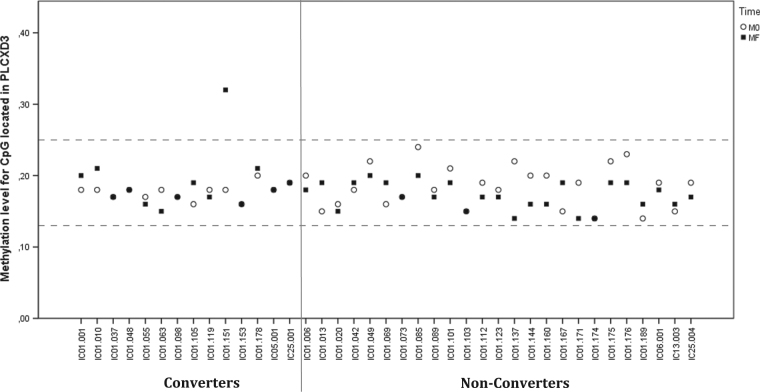
Example of an outlier detection (cg14099514 in PLCXD3 gene). Methylation level of this CpG is quite stable (comprised between 14% and 24% whatever the clinical status and the time of assessment) excepted for one individual considered as an outlier (IC01.151), which shows deviation only after the psychotic transition (methylation level = 32% at MF)

Variance analyses led to the detection of many outliers in converters and non-converters at M0 and MF. Individual variability appeared highly frequent. According to the *F*-test, 220 and 6007 CpGs were significant in converters and non-converters respectively (including 156 CpGs common to both converters and non-converters). The high number of CpG outliers in non-converters may index processes that are not related to conversion to psychosis. It can also refer to processes normally active in individuals in a longitudinal exploration. In our context, the large number of CpG outliers in non-converters could also be due to the larger size of this group compared to the converters group. Filtering criteria only retained for further analysis CpGs with a longitudinal increase in variance in converters that did not overlap with significant CpGs found in non-converters. Twenty-five CpGs were selected at this point (Table 2). Raw data were extracted for each converter and difference with the group median is given in Table 3. When considering individual deviations from the group median which are >10%, 12 CpGs were selected as private epimutation loci plausibly affecting psychosis onset. For example, Fig. 2 provides a graphical representation of a CpG located in phosphatidylinositol specific phospholipase C X domain containing the 3 (PLCXD3) gene (cg14099514, chr5:41510519). Four individuals did not show any epimutation, whereas five individuals displayed one private epimutation. Four individuals had two private epimutations and one individual had three private epimutations (16 epimutations in 14 individuals). Four CpGs were identified as dysregulated in two individuals with a similar trend over time. One CpG (cg12053442) underwent demethylation and three CpGs (cg19041132, cg01558909, and cg14993491) underwent hypermethylation. All these CpGs are more likely to be associated with psychotic conversion. We examined whether they might be related to a specific biological network. Over-representation analysis of the CpG loci was performed. Genes from the sphingolipid signaling pathway were hyper-represented with Ras-related C3 botulinum toxin substrate 1 (RAC1) and Sphingosine kinase 1 (SPHK1). Interestingly, one individual (IC01.055—Table 3) had a longitudinal increase of methylation in both RAC1 and SPHK1, suggesting common epigenetic regulation for these two genes.

**Table 2:** The 25 variable CpGs identified in converters as clinically relevant epimutations

CpG	*p*-value of *F*-test (FDR)	Genes
cg18796523	8.46E−04	*ALG11; ATP7B*
cg04087237	4.41E−02	*CACNB4*
chr16:50203861	9.91E−03	—
cg13303475	1.10E−02	*NT5DC3*
cg01918706	4.37E−02	*UBE2T*
cg01657694	3.09E−02	*KLHL22*
cg13694867	2.54E−02	*SIM2*
cg05168033	1.24E−03	*EFEMP1*
cg26952925	1.16E−02	*ADCY9*
cg13562542	5.93E−03	*GPR27; EIF4E3*
cg17364044	3.10E−02	*PELI1*
cg26447413	2.02E−02	*GAS1*
cg11265160	1.48E−02	*TMEM132C*
cg21849932	1.87E−02	*LIME1*
cg04364540	4.27E−03	*FAM160B1*
cg14099514	1.02E−02	*PLCXD3*
cg19176559	3.46E−03	—
cg19041132	6.22E−07	*SPHK1*
cg12053442	2.85E−02	—
cg01558909	1.85E−05	*HBM*
cg14993491	4.54E−03	*PCSK9*
cg13408519	3.24E−06	—
cg12604181	1.04E−09	*CTSH*
cg18404925	1.50E−07	*RAC1*
cg13978347	1.08E−04	*ASTN2*

Significance in *F*-test are corrected by FDR

**Table 3:** Representation of the private epimutations (lines) in each converter (columns, *n* = 14)

Genes	CpG	IC01.001	IC01.010	IC01.037	IC01.048	IC01.055	IC01.063	IC01.098	IC01.105	IC01.119	IC01.151	IC01.153	IC01.178	IC05.001	IC25.001
Before transition															
* ALG11; ATP7B*	cg18796523	0.00	0.00	0.00	0.00	0.00	0.00	0.00	0.00	0.00	0.00	0.00	0.00	0.00	0.00
* CACNB4*	cg04087237	0.00	0.00	0.00	0.00	0.01	0.00	0.00	0.00	0.00	0.00	0.01	0.00	0.00	0.00
—	ch.16,50203861R	0.00	0.00	0.00	0.00	0.00	0.00	0.00	0.00	0.00	0.00	0.00	0.00	−0.01	−0.01
* NT5DC3*	cg13303475	0.00	0.00	0.00	0.00	0.00	0.00	0.00	0.01	0.00	0.00	0.00	0.00	−0.01	0.00
* UBE2T*	cg01918706	0.00	0.00	0.00	0.00	0.00	0.00	0.01	0.01	0.00	0.00	0.00	0.00	−0.01	0.01
* KLHL22*	cg01657694	0.00	0.01	0.00	0.00	−0.01	0.00	0.00	0.00	0.00	0.00	0.01	0.00	−0.01	0.00
* SIM2*	cg13694867	0.00	−0.01	0.00	0.00	0.00	0.00	0.00	0.01	0.01	0.01	0.00	0.00	0.00	0.00
* EFEMP1*	cg05168033	0.00	0.00	0.00	0.00	0.00	0.00	−0.01	0.00	0.00	0.00	0.00	0.00	−0.01	0.00
* ADCY9*	cg26952925	0.00	−0.01	0.01	0.00	0.00	0.00	0.01	0.00	0.00	0.00	−0.01	0.00	0.00	0.01
* GPR27; EIF4E3*	cg13562542	0.01	0.00	0.00	0.00	−0.01	0.00	0.00	0.01	0.00	0.00	0.00	0.00	0.00	0.00
* PELI1*	cg17364044	−0.01	0.01	−0.01	0.00	0.01	0.00	−0.01	0.00	0.00	0.01	−0.01	0.00	0.00	0.00
* GAS1*	cg26447413	0.00	−0.01	−0.01	0.01	0.00	0.00	−0.01	0.00	0.00	0.01	0.00	0.00	0.00	0.00
* TMEM132C*	cg11265160	0.00	−0.01	−0.01	0.01	−0.01	0.00	0.00	0.02	0.00	0.00	0.00	0.00	0.00	−0.01
* LIME1*	cg21849932	0.00	0.00	0.00	0.00	0.00	0.00	0.03	0.00	−0.01	0.01	0.00	0.00	−0.01	0.01
* FAM160B1*	cg04364540	−0.01	0.00	0.01	0.00	0.02	0.01	0.00	0.00	0.01	0.00	0.01	−0.01	−0.01	−0.01
* PLCXD3*	cg14099514	0.00	0.00	−0.01	0.00	−0.01	0.00	−0.01	−0.02	0.00	0.00	−0.02	0.02	0.00	0.01
—	cg19176559	−0.01	−0.01	0.01	0.00	−0.02	0.01	−0.01	−0.01	0.01	0.01	0.01	−0.01	0.00	0.01
* SPHK1*	cg19041132	0.00	0.00	0.00	0.00	0.00	0.00	0.00	0.01	0.00	0.00	0.00	0.00	0.00	0.00
—	cg12053442	−0.01	0.00	−0.01	0.02	0.01	0.01	−0.03	0.00	−0.01	0.02	−0.01	0.00	0.01	−0.01
* HBM*	cg01558909	0.00	−0.01	−0.01	0.00	0.01	0.01	−0.01	0.03	0.01	0.02	−0.01	0.01	0.00	−0.01
* PCSK9*	cg14993491	−0.02	0.01	0.02	−0.02	0.01	0.02	−0.03	−0.01	0.05	0.03	−0.04	−0.02	0.02	−0.02
—	cg13408519	0.02	0.00	−0.01	0.02	0.00	0.02	0.00	−0.02	−0.01	0.02	0.01	0.00	−0.01	−0.01
* CTSH*	cg12604181	0.00	0.01	0.00	0.00	0.01	0.00	0.00	0.02	0.00	0.00	0.01	0.00	0.00	−0.01
* RAC1*	cg18404925	0.00	0.00	−0.01	−0.01	0.01	0.01	−0.02	0.00	0.01	−0.03	0.01	0.00	−0.01	−0.03
* ASTN2*	cg13978347	−0.01	−0.03	0.00	0.06	−0.02	0.02	0.01	0.00	0.03	0.01	0.06	−0.04	0.00	0.00
After transition															
Antipsychotic introduction		×	×	×	×		×								×
* ALG11; ATP7B*	cg18796523	0.00	0.00	0.01	0.00	0.00	0.00	0.00	0.02	−0.01	0.03	0.01	0.00	0.00	0.00
* CACNB4*	cg04087237	0.04	0.02	0.00	−0.01	0.00	0.00	−0.01	0.01	0.00	0.00	0.00	0.00	0.00	−0.01
—	ch.16.50203861R	0.00	−0.01	0.00	−0.01	0.01	0.00	0.00	0.00	0.00	0.06	0.00	0.01	0.00	0.00
* NT5DC3*	cg13303475	0.00	0.00	0.00	0.00	0.01	−0.01	−0.01	0.00	0.00	0.00	0.00	0.06	−0.01	0.00
* UBE2T*	cg01918706	0.00	0.00	0.01	0.00	0.00	0.00	0.06	0.00	0.00	0.00	0.01	0.00	0.00	−0.01
* KLHL22*	cg01657694	0.03	0.01	0.00	0.01	0.00	0.00	0.00	0.06	0.00	0.00	0.00	0.01	−0.01	−0.01
* SIM2*	cg13694867	0.02	0.00	−0.01	0.01	−0.01	−0.02	0.00	0.00	−0.03	−0.01	0.00	−0.01	0.00	0.05
* EFEMP1*	cg05168033	0.04	0.01	0.00	0.00	0.00	−0.01	−0.01	0.01	0.00	0.01	0.00	0.01	0.00	0.06
* ADCY9*	cg26952925	0.00	0.00	0.00	0.01	0.00	−0.01	0.00	0.01	0.00	0.01	0.00	−0.01	0.00	0.08
* GPR27; EIF4E3*	cg13562542	0.07	0.00	−0.02	0.01	0.00	0.00	−0.01	−0.01	0.01	0.00	0.00	0.01	−0.01	0.01
* PELI1*	cg17364044	0.00	−0.02	−0.01	−0.01	−0.02	0.00	0.00	0.10	0.00	0.01	0.01	−0.01	−0.01	0.00
* GAS1*	cg26447413	−0.01	0.01	0.00	0.01	−0.02	−0.01	−0.01	−0.02	0.01	0.10	−0.01	0.00	0.02	0.00
* TMEM132C*	cg11265160	0.01	0.08	0.00	0.01	−0.02	−0.05	−0.04	−0.01	0.00	0.00	−0.06	0.00	0.01	0.00
* LIME1*	cg21849932	−0.01	0.01	0.00	0.00	0.00	0.01	**0.13**	0.00	0.00	0.00	0.00	0.00	−0.01	0.00
* FAM160B1*	cg04364540	0.00	−0.01	−0.01	0.00	0.01	0.00	0.00	0.01	−0.01	0.00	0.00	**0.15**	−0.02	0.00
* PLCXD3*	cg14099514	0.02	0.03	−0.01	0.00	−0.02	−0.04	−0.01	0.00	−0.01	**0.14**	−0.03	0.02	0.00	0.00
—	cg19176559	0.04	−0.03	0.00	0.00	−0.01	−0.02	**0.15**	0.01	0.00	0.01	−0.02	0.01	0.00	0.02
* SPHK1*	cg19041132	0.00	0.00	0.00	0.02	**0.12**	−0.01	0.00	0.01	0.00	0.00	−0.01	**0.13**	−0.01	0.00
—	cg12053442	−0.01	−0.01	0.01	−0.01	0.00	0.00	−0.01	*−0.15*	0.01	0.00	0.02	0.00	0.01	*−0.11*
* HBM*	cg01558909	**0.20**	0.00	−0.01	−0.01	0.01	**0.29**	0.07	0.00	−0.02	0.03	0.01	0.00	−0.01	0.00
* PCSK9*	cg14993491	−0.04	**0.25**	0.02	−0.05	−0.04	**0.36**	−0.05	−0.02	−0.01	0.02	−0.01	0.01	0.01	0.02
—	cg13408519	−0.01	0.01	−0.01	0.01	−0.02	−0.01	0.00	0.00	0.00	0.00	0.03	0.01	−0.01	**0.46**
* CTSH*	cg12604181	0.00	0.00	0.00	**0.54**	0.09	0.01	0.01	0.00	−0.01	0.00	0.01	−0.01	−0.02	0.00
* RAC1*	cg18404925	−0.03	−0.01	0.00	0.06	**0.63**	0.03	−0.01	−0.01	0.00	0.03	0.02	0.01	−0.02	−0.02
* ASTN2*	cg13978347	0.04	−0.02	0.02	−0.01	0.01	0.01	0.03	0.02	−0.05	−0.04	0.02	*−0.71*	−0.03	−0.01

The value in each case correspond to the deviation from the individual *β*-value to the group median: relative hypermethylation is in bold and relative hypomethylation in italics

## Discussion

Understanding the molecular mechanisms associated with the emergence of psychosis requires detecting, not only inter-group differences as we reported previously in epigenetic^11^ or transcriptomic^20^ parameters, but also individual variability. This is the first study describing the longitudinal methylomic variability in association with conversion to psychosis using new statistical approaches to detect the changes at a more individualized ‘private’ level.

First, two VMPs were identified. VMPs display many longitudinal methylation changes and are supposed to tag regions with methylomic instability. One CpG was located in MACRO Domain Containing 2 (MACROD2) gene. It encodes an evolutionarily conserved macro domain protein whose significant role in multiple biological processes includes DNA repair, transcriptional activation, and repression. Genetic variants in MACROD2 had been reported in association with schizophrenia^21^, autism^22^, and attention-deficit hyperactivity disorder^23^. Second, using variance analysis to detect outliers, a dozen of private epimutations were identified, including two genes (RAC1 and SPHK1) from the sphingolipid signaling pathway. RAC1 regulates a diverse array of cellular events, including cell growth control, cytoskeletal reorganization, and the activation of protein kinases. The Rac1 protein is activated by the *N*-methyl-d-aspartate receptor (NMDAR) and is important for disrupted in schizophrenia 1 (DISC1) function in the maintenance of spine morphology and function^24^. Both NMDAR and DISC1 functions are strongly related to schizophrenia^25–27^. Moreover, lower Rac1 levels were reported in the post-mortem dorsolateral prefrontal cortex in schizophrenia^28^. Hypoexpression of RAC1 is consistent with our findings (hypermethylation). SPHK1 catalyzes the phosphorylation of sphingosine to form sphingosine-1-phosphate (S1P), a lipid mediator. Intracellularly, S1P regulates proliferation and survival, and acts as an extracellular ligand for cell surface G protein-coupled receptors. The protein and its S1P product have a key role in tumor necrosis factor-alpha signaling and in the nuclear factor-kappa-B activation pathway. This role is crucial in inflammatory, anti-apoptotic, and immune processes. Interestingly, SphK1 activation is involved in the regulation of lipopolysaccharide induced neuro-inflammation^29^, and its inhibition has a major role in caspase-dependent apoptotic neuronal death^30^. In summary, the three newly identified genes (MACROD2, RAC1, and SPHK1) are related to brain functions and have plausibility for involvement in the pathophysiology of psychosis. Increased methylation variability in some UHR individuals may have triggered critical changes in gene networks containing these genes and accelerated conversion to psychosis. Private epimutations appeared neither necessary nor sufficient to explain conversion to psychosis. Indeed, some converters did not have any epimutations whereas some non-converters displayed one or several epimutations. We suggest that private epimutations could trigger the onset of psychosis by acting in synergy with more common epigenetic changes, as those previously reported^11^. This interaction could account for the heterogeneity of the pathological course and for the clinical presentation.

Changes in the epigenome are directly attributable to individual and combined effects of genetics, environment, or stochasticity. It has been hypothesized that stochasticity has a role in epigenetic variability. Indeed, a stochastic process has some elements of noise or randomness resulting in different outcomes, given similar initial conditions. In epigenetics, stochasticity refers to a process where epigenetic factors can ‘mutate’ in the absence of any detectable environmental influence, e.g., no fidelity during DNA methylation replication^10^. However, the part attributable to stochasticity is hardly quantifiable. One aspect of epigenetic stochasticity is hypervariable methylation. We call epigenetic drift the spontaneous changes in cell methylome over time. Experimental evidence for epigenetic drift in humans comes from longitudinal studies of monozygotic twin methylomes^31–33^. For example, methylation across the promoters of three genes (dopamine receptor 4, serotonin transporter, and X-linked monoamine oxidase) was quantified in a large number of monozygotic and dizygotic twins at 5 and 10 years of age^34^. This study reported that even genetically identical organisms show evidence of epigenetic drift with age. Previous twin studies also suggested that intra-pair methylomic changes might reflect complex, cumulative phenomena including stochastic effects occurring over decades of life, from birth to the time of molecular investigation. Our study bears similarities to this approach by controlling for the genetic variability using intra-individual comparison, with samples before and after disease onset. Though samples provided by discordant monozygotic twins are rare, our approach could be developed on a larger scale. Moreover, the longitudinal assessment explores the epigenetic drift occurring during the onset of a disease, whereas epigenetic drift in twins could be slower and less related to pathophysiology.

Some limitations of the present study should be mentioned. First, sample size is limited, which needs future replication. Second, we used a methylation chip that offers a limited coverage, compared to the number of CpGs in the genome. Thus, potential variable probes located in uncovered genomic regions have been omitted. Third, the extent to which the present peripheral marker-based findings reflect methylation processes in the brain cannot be firmly established. Yet, it is noteworthy that concordant blood and brain methylation levels were reported^35^. Finally, we cannot exclude the possibility that in some individuals, methylation changes could be related to antipsychotic treatment initiation. Changes in the HBM, CTSH, and PCSK9 genes notably occur only in individuals with antipsychotic treatment initiation. On the contrary, changes in individuals without psychotropic drug initiation, such as RAC1 and SPHK1, are unlikely to be due to treatment. Finally, even though the results based on data from non-converters were ruled out, we cannot assert with certainty that the methylation variability observed in converters is specific to the emergence of psychosis.

In summary, few statistical methods have been developed to investigate epigenetic stochasticity. We propose to generalize two methods applicable to methylomic beadchips, namely (i) searching for CpG sites with large variability by comparing medians, and (ii) searching for private epimutations by comparing variances to identify outliers. Though our approach enables detection of rare events and analysis of individual characteristics, developing new statistical approaches will be useful to address the heterogeneity of complex diseases. The present results support the theoretical assumption that DNA methylation variability may contribute to the complex, heterogeneous pathophysiology of conversion to psychosis.

## Electronic supplementary material

R scripts
